# Measurement of renal function: Should cystatin C be more widely used for people with diabetes?

**DOI:** 10.1111/1753-0407.13534

**Published:** 2024-01-28

**Authors:** Christian W. Mende, Zachary Bloomgarden

**Affiliations:** ^1^ Department of Nephrology University of California San Diego California USA; ^2^ Department of Medicine Mount Sinai School of Medicine New York New York USA

Chronic kidney disease (CKD) affects about 10% of the adult population worldwide^1^ and 14% of the adult population of the United States.^2^ CKD is diagnosed by either of two criteria being present for 3 months or longer: measured renal function using the recently updated race free Chronic Kidney Disease Epidemiology (CKD‐EPI) 2021 formula with an estimated glomerular filtration rate (eGFR) <60 mL/min/1.73 m^2^ and/or albuminuria with urine albumin/ creatinine ratio(UACR) >30 mg/g.^3^ Analysis of data from the US National Health and Nutrition Examination Survey through 2012 showed evidence of CKD based on eGFR and/or UACR in approximately 40% of persons with type 2 diabetes (T2D) and in approximately 60% of those with T2D age ≥65,^4^, ^5^ and the reported CKD prevalence among persons with T2D globally is approximately 50%.^6^ Both eGFR and UACR are important as risk markers for atherosclerotic cardiovascular disease, heart failure, and mortality.^7^ CKD must also be taken into account in drug dosing for medications with predominantly renal excretion, recognizing that glomerular filtration is only one part of the mechanism of drug elimination by the kidneys, which also involves tubular secretion and tubular reabsorption.^8^ Renal function measurement in clinical practice is typically accomplished by assessing estimated glomerular function or eGFR ml/min/1.73 m^2^ using serum creatinine (eGFR_creat_).

The eGFR_creat_ shows day‐to‐day variability of 4%–10% in healthy individuals and of 5%–15% in people with CKD.^9^, ^10^ Both high and low creatinine generation have major impact on the accuracy of eGFR_creat_. A high protein diet, including one with protein supplements, and drugs reducing tubular creatinine excretion, such as trimethoprim, H2 blockers, fenofibrate, and ritonavir and other HIV drugs, lead to falsely higher eGFR_creat_. On the other hand, low creatine levels from a vegetarian diet, limb amputation, and states of sarcopenia including malnutrition, cirrhosis, and malignancies will be associated with an incorrectly lower eGFR_creat_.^11^

Cystatin C has been known for over 60 years^12^ and the Food and Drug Administration approved a cystatin C assay in 2001 for use in estimating the GFR. Cystatin C, a member of a family of inhibitors of thiol proteinases, is generated by all nucleated cells. It is a low molecular weight protein that is filtered by the renal glomeruli and completely reabsorbed and metabolized in the proximal tubule with none excreted in the urine.^13^ It is not affected by race or muscle mass, although levels are increased by obesity, inflammation, and thyroid disease and by steroid therapy, chemotherapy, and smoking.^11^, ^14^ These features make cystatin C an attractive alternative and addition to creatinine in the estimation of GFR (eGFR_Cystatin C_). A systematic review of use of eGFR_Creat_ vs eGFR_Cystatin C_ as kidney biomarkers showed the latter to be as accurate and, in most studies, superior for determination of drug dosing.^15^

For these reasons in 2012 Kidney Disease: Improving Global Outcomes recommended the use of cystatin C as an alternative and addition to eGFR_creat_ in confirming the diagnosis of CKD. Discrepancies between cystatin C and creatinine eGFR were greatest for eGFR Stage 3 (30–60 mL/min) in a general population.^16^

The accepted gold standard for eGFR measurement is the measurement of iothalamate plasma clearance after a bolus injection. A study of persons with T2D with preserved renal function using an alternate research method for eGFR measurements, plasma inulin clearance, noted that eGFR_creat_ underestimated measured GFR by 46%, suggesting the importance of developing alternative approaches.^17^ In a population of persons with type 1 diabetes and a wide range of GFR levels, eGFR estimated from cystatin C levels was more reliable than that estimated from creatinine.^18^ Furthermore, a study of changes in serum cystatin C over time showed that persons with T2D with higher baseline cystatin C levels and greater rate of increase in these levels have increased risk of subsequent CKD development.^19^

In a phase 3 trial in 4745 T2D patients treated with dapagliflozin with eGFR_creat_ between 30 and 60 mL/min (Stage 3 CKD), the use of eGFR_Cystatin_ reclassified 66% of patients as having eGFR >60 mL/ min.^20^ The use of eGFR_Cystatin_ improved the categorization of CKD and risk estimation for all‐cause mortality and ESKD in a meta‐analysis of 11 population studies with 90 750 participants using both cystatin C and creatinine eGFR, showing in particular that, for CKD stage3a (eGFR_creat_ 45–59 mL/min), 42% were reclassified using eGFR_Cystatin_ as having an eGFR >60 mL/min.^16^ In a pooled population of 4050 persons from 12 trials, a new eGFR equation with combined use of creatinine and cystatin C performed best in assessing renal function.^21^ Importantly, when eGFR_Creat_ vs eGFR_Cystatin C_ are highly discordant, the new eGFR equation leads to greater accuracy in characterizing a given individual.^22^

Additionally, with significant weight loss, as occurs following bariatric surgery and with incretin‐based diabetes treatment approaches, approximately 25% of lost weight is due to loss of skeletal muscle.^23^ The use of eGFR_creat_ in this setting might overestimate renal function – although this has not been directly investigated. Although the cost of measurement of cystatin C is somewhat greater than that of measurement of creatinine,^11^ given the importance for persons with diabetes of accurately arriving at the correct renal function and classification of CKD and its cardiovascular risk, use of cystatin C along with creatinine in estimating eGFR appears appropriate.
